# The impact of family interventions on communication in the context of anxiety and depression in those aged 14–24 years: systematic review of randomised control trials

**DOI:** 10.1192/bjo.2023.545

**Published:** 2023-08-29

**Authors:** Alex Lloyd, Amy Broadbent, Edmund Brooks, Karen Bulsara, Kim Donoghue, Rouhma Saijaf, Katie N. Sampson, Abigail Thomson, Pasco Fearon, Peter J. Lawrence

**Affiliations:** Department of Clinical, Educational and Health Psychology, Division of Psychology and Language Sciences, University College London, UK; Member of the Young People's Advisory Group; Member of the Parents and Carers' Advisory Group; National Collaborating Centre for Mental Health, The Royal College of Psychiatrists, London, UK; Department of Experimental Psychology, University of Oxford, UK; Department of Clinical, Educational and Health Psychology, Division of Psychology and Language Sciences, University College London, UK; Department of Psychology, University of Cambridge, UK; and Developmental Neuroscience Unit, Anna Freud Centre, London, UK; School of Psychology, University of Southampton, UK

**Keywords:** Anxiety or fear-related disorders, depressive disorders, psychosocial interventions, cognitive–behavioural therapies, randomised controlled trial

## Abstract

**Background:**

The ability to communicate is integral to all human relationships. Previous research has specifically highlighted communication within families as both a risk and protective factor for anxiety disorders and/or depression. Yet, there is limited understanding about whether communication is amenable to intervention in the context of adolescent psychopathology, and whether doing so improves outcomes.

**Aims:**

The aim of this systematic review was to determine in which contexts and for whom does addressing communication in families appear to work, not work and why?

**Method:**

We pre-registered our systematic review with PROSPERO (identifier CRD42022298719), followed Preferred Reporting Items for Systematic Reviews and Meta-Analyses guidance and assessed study quality with the Risk of Bias 2 tool.

**Results:**

Seven randomised controlled trials were identified from a systematic search of the literature. There was significant heterogeneity in the features of communication that were measured across these studies. There were mixed findings regarding whether family-focused interventions led to improvements in communication. Although there was limited evidence that family-focused interventions led to improvements in communication relative to interventions without a family-focused component, we discuss these findings in the context of the significant limitations in the studies reviewed.

**Conclusions:**

We conclude that further research is required to assess the efficacy of family-focused interventions for improving communication in the context of anxiety and depression in those aged 14–24 years.

Anxiety disorders and depression are common mental health problems affecting approximately 3.6% and 4.4% of the global population, respectively.^1^ A significant proportion of these common mental health problems emerge before or during adolescence (51.8% for anxiety disorders and 11.5% for mood disorders^2^). Effective pharmacological and psychological treatments exist,^3,4^ but their effectiveness is only moderate and do not work for everyone.^5^ Furthermore, ‘how’ these treatments work and, more specifically, what their ‘active ingredients’ are, remains relatively unclear. The focus of our insight review is on improving communication in families as an active ingredient in the effective treatment of anxiety disorders and/or depression in young people aged 14–24 years. Focus on family communication has historically been rooted in biosocial models of psychopathology, which emphasise that mental health problems are embedded in the individual's social context.^6^ On these accounts, relationships, and the communication between individuals within those relationships, are implicated in the aetiology and possible treatment of psychopathology, including anxiety disorders and depression.

Communication is an essential component of human social functioning. To define communication within a family context, we draw on seminal work by Fitzpatrick and Ritchie,^7^ who argue that family communication can be understood within the dimensions of ‘openness and emotional accessibility’ and ‘structural traditionalism’. For the purposes of this review, we focus on openness and emotional accessibility, which describes the extent to which family members exhibit ‘openness in deployment of emotional resources, receptivity to new information, and shared responsibility for coping with daily emotion and social crises’.^7^ Within this framework, family communication can be measured as the extent to which family members reciprocate feelings to one another, solve problems together and are open to new information without it causing conflict.^7^ Indeed, psychometric studies that have developed scales to measure family functioning incorporate the definitions established in this early work.^8^

Given the ubiquity of communication in human relationships, it has been argued that negative patterns of communication can contribute to the onset of mental health problems, as they exacerbate risk factors that may predispose the individual to psychopathology.^6^ Longitudinal^9^ and cross-sectional evidence^10–12^ suggests that open and respectful communication between parents and their adolescent offspring reduces the risk common mental health problems. Furthermore, influential developmental models of anxiety and depression implicitly implicate communication. For example, Bowlby's attachment theory assumes that ‘internal working models’ of attachment are generated in the context of goal-corrected partnership with primary attachment figures, where communication of emotional needs is central.^13^ Similarly, cognitive–behavioural models assume that experiences in key relationship influence the development of schemas that influence cognition and emotion. These schemas embed interpersonal experiences, within which communication is likely to be central. Psychological abuse, which is strongly associated with risk for psychopathology, largely involves forms of highly pathological communication on the part of the perpetrator.^14^

Family systems theory provides arguably the most comprehensive theoretical framework for understanding the role of communication in psychopathology. The theory takes a systemic approach to mental health, placing emphasis on the dynamics within the family system rather than the individual themselves.^15^ Family systems comprise individuals within the family (e.g. parents or siblings) as well as wider networks that influence family members, including peers, grandparents, colleagues and other environmental factors such as socioeconomic status and school climate.^16,17^ Within these systems are further subsystems, such as parent–child dyads or sibling dyads. The relationships between individuals within families are influenced in a cyclical manner, such that positive interactions perpetuate positive patterns, whereas negative interactions perpetuate negative patterns. Communication, which we restrict to verbal modes of interaction in the current review, is the means by which patterns of interactions are created, maintained or perpetuated in the family systems framework.^18^

Consistent with the family systems framework, empirical evidence has demonstrated that family climate and communication styles are a predictor of depression and anxiety in adolescence.^19–22^ Indeed, one study found that a lack of family cohesion was associated with increased risk for any mental health disorder (including depression and anxiety).^23^ The mechanisms that link poor family communication to adolescent anxiety and depression are likely to be multifaceted and heterogeneous across individuals.^24^ For example, poor communication patterns can reduce adolescents’ problem-solving capabilities,^21^ which may heighten vulnerability to depression via increased rumination.^25^ Further, familial discord resulting from poor communication can remove an important support system for the adolescent, as parental support is associated with reduced anxiety and depressive symptoms.^26,27^ In contrast, good communication within families has also been demonstrated to be a protective factor in adolescent mental health. For example, positive family communication predicts higher self-esteem in adolescence, which is negatively associated with symptoms of anxiety and depression.^28,29^ Moreover, parent–adolescent communication is positively associated with well-being and life satisfaction, but negatively associated with internalising and externalising symptoms.^30^ Together, these empirical studies suggest that poor family communication is an important and multifaceted risk factor for psychopathology in adolescence.

Another route through which poor communication may increase the risk of poor mental health outcomes is by acting as a barrier to treatment. Parents are often key figures in supporting young people's engagement with mental health services, and the quality of communication between young people and their parents is therefore likely to be crucial in accessing treatment and engaging with the support offered. Parents are often involved in shared decision-making about interventions their child receives,^31^ and qualitative work has identified familial conflict as a barrier to parents and adolescents collaboratively seeking mental health treatments.^32^ Further, encouraging parental support for therapeutic treatment improves adolescents’ attendance and adherence to these interventions,^33^ which may be partly because of the dependence adolescents have on parents for accessing support (e.g. facilitating travel to the treatment site^34^). As such, good family communication may have general mental health benefits and may enhance the efficacy of other interventions; and, where problems exist, it may be necessary to address family communication before other interventions can be successfully implemented (akin to the need to treat high blood pressure before conducting major surgery).

## Rationale for the current study

Despite evidence that family communication is a multifaceted risk and protective factor for depression and anxiety disorders, there is limited understanding of whether interventions can improve communication within families, and whether this has positive implications for adolescents’ mental health. The aim of the current systematic review was to determine in which contexts, and for whom, addressing communication in families appears to work, not work and why. We also aimed to examine factors that may moderate the efficacy of these family-focused interventions (e.g. gender, culture, structure of the family unit, family knowledge of anxiety and depression). Throughout the review, we have worked with experts by experience to co-produce the views presented in this article.

## Method

### Advisory group involvement

We created two groups of experts by experience: a Young People's Advisory Group (YPAG) and a Parents and Carers' Advisory Group (PCAG). The YPAG comprised four regular members (aged 16–23 years) and met four times for 90 min. The PCAG comprised three regular members and met three times for 90 min. There was one joint 90 min meeting between the PCAG and YPAG. YPAG and PCAG members received £20 per hour for attending meetings, and £15 per hour for work between meetings.

Following our first YPAG meeting, we pre-registered our systematic review with the International Prospective Register of Systematic Reviews ( PROSPERO; identifier CRD42022298719) and followed Preferred Reporting Items for Systematic Reviews and Meta-Analyses guidelines. The PICO framework (population, intervention, control and outcomes) was used to establish inclusion/exclusion criteria and search strategy.

### Eligibility criteria

A study was eligible for inclusion if (a) it was a randomised controlled trial; (b) participants were aged between 14 and 24 years; (c) participants had a diagnosis of anxiety disorder and/or depression, as established by DSM or ICD diagnostic criteria or a validated scale; (d) the study intervention involved family members as well as the young person themselves, and family was broadly defined as parents, carers or guardians, siblings or extended relatives; (e) the study included a control group (e.g. treatment as usual or waitlist); (f) the study reported outcomes relating to family communication (improving communication, specifically verbal communication, was defined in terms of reductions in critical, hostile or isolating interactions, or increases in supportive or warm verbal interactions); and (g) the study was available in full text in the English language in a peer-reviewed journal.

### Search strategy

EMBASE, Medline and PsycINFO were searched via the OVID interface, and the Cochrane Central Register of Controlled Trials database was also searched. Searches were completed on 20 January 2022, combining MeSH and free-text terms for our population (children and adolescents aged 14–24 years), intervention (family-focused interventions for common mental disorders), outcome (family communication) and study design (randomised controlled trials). There were no restrictions on the publication date. The reference lists and citations of included articles were also searched. The full search strategy is presented in Supplementary Table 1 available at https://doi.org/10.1192/bjo.2023.545.

### Study selection and data extraction

Identified articles were imported into the online software Rayyan (Rayyan Systems, Cambridge, Massachusetts, USA; http://rayyan.qcri.org),^35^ and duplicates were removed. Two reviewers (K.N.S. and K.D.) independently screened the titles and abstracts against the eligibility criteria with Rayyan. Any disagreements for inclusion were discussed by K.N.S. and K.D., and consensus reached. Full texts were then retrieved and screened for eligibility independently by K.N.S. and K.D., using a form developed in Microsoft Excel (2019, for Windows). Any disagreements for inclusion were discussed and resolved between the two reviewers. A data extraction form (available from P.J.L.) was created in Microsoft Excel and data was extracted independently by K.N.S. and K.D. The following data were extracted:

- study characteristics: author, year, country, study design features, recruitment method, allocation method, inclusion/exclusion criteria, follow-up period and sample size;
- participant characteristics: age, gender, ethnicity, diagnosis and method of ascertainment;
- intervention: description of intervention, duration of intervention, number and length of sessions and family involvement (percentage of total time);
- comparison: comparator intervention and characteristics of control group;
- outcomes: data collection points, primary family communication outcome and method of measurement, analysis method, main results, missing data and loss to follow-up;
- potential moderators: gender, culture, structure of the family unit, family knowledge of anxiety and depression, duration of the intervention and percentage of family involvement;
- general: limitations as identified by study author(s) and funding.

All included studies were assessed for risk of bias with the Cochrane Risk of Bias 2 tool.^36^ The two reviewers assessed studies independently with the Risk of Bias 2 tool, and consensus was reached through discussion.

Data extraction began on 31 January 2022.

### Data synthesis

The data available from the included studies were insufficient to support a robust or meaningful meta-analysis. Improving family communication was highly heterogeneous across studies with regards to how interventions attempted to alter patterns of communication and how communication was measured. Therefore, the results of the systematic review were synthesised narratively.

## Results

### Summary of studies

**Fig. 1:**
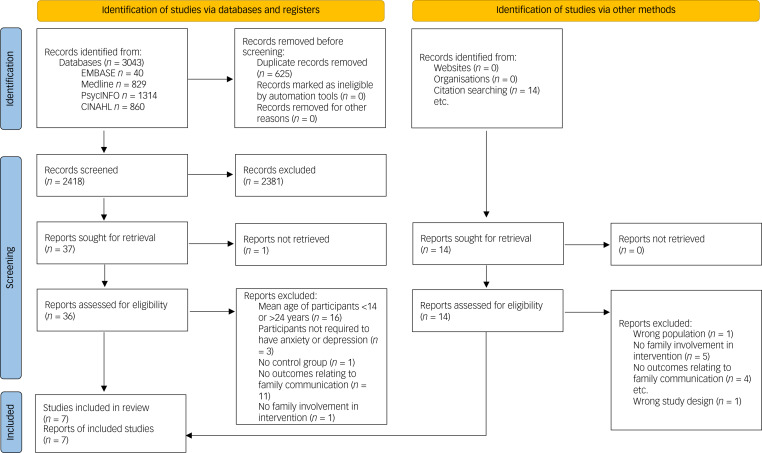
Preferred Reporting Items for Systematic Reviews and Meta-Analyses flow chart of systematic literature search.

Seven studies met our inclusion criteria (see Fig. 1). These studies included 440 participants in total, with a mean age of 15.71 years (note, Bernal et al^37^ did not report participants’ mean age and was excluded from this calculation). Depression was the primary outcome measure in the studies reviewed; all studies measured depressive symptoms.^37–43^ Five studies additionally measured whether participants met the diagnostic criteria for major depressive disorder (see Table 1).^37,41–43^ Five studies recruited participants from the community^37–39,41^ and two studies recruited participants from out-patient clinics.^42,43^

**Table 1:** Details of the studies examining family communication in context of anxiety and depression included in the current review

Study	Population	Intervention	Comparator	Outcome	Notes
Dietz et al,^38^ 2014, three-arm RCT based in the USA, lasting 24 months	63 adolescents with mean age 15.6 (s.d. 1.3) years, meeting DSM-III-R diagnostic criteria for MDD Gender (% female): SBFT group 75%, CBT group 66.9%, NST group 83.3% Ethnicity (% minority status): SBFT group 10%, CBT group 24%, NST group 11.1% Socioeconomic status (Hollingshead mean, s.d.): SBFT group 41.1 (10.3), CBT group 38.7 (11.6), NST group 38.9 (18.1) 20.6% of participants had comorbid disruptive behaviour disorder 14.3% of participants had comorbid anxiety disorder	SBFT: *n =* 20 Combined aspects of functional family therapy, intended to clarify concerns and identify dysfunctional patterns of family interaction, and a behavioural family systems approach, aimed at teaching communication and problem-solving skills to reduce family conflict 12–16 weekly sessions Family involved for all sessions. Up to 25% of total treatment time (4 h) could be allocated to patient individually without family	CBT: *n = 25* Intervention focused heavily on cognitive restructuring, also utilising behavioural activation, and taught problem-solving skills on a case-by-case basis NST: *n =* 18 Focused on providing support, aiding the adolescent in affect identification and expression of feelings, and discussing patient-initiated options for addressing problems. NST therapists relied on accurate empathy and reflexive listening, and refrained from giving advice, setting limits or teaching specific skills 12–16 weekly sessions Up to 25% of total treatment time (4 h) could be allocated to family involvement	Adolescent interpersonal behaviour (adolescent involvement, adolescent problem-solving and dyadic conflict) coded from videotapes of 10-min mother–adolescent interactions, in which two issues causing conflict in the relationship were discussed CBT (B = 0.41, 95% CI 0.29–1.67, *t* = 2.85, *P* = 0.006) and SBFT (B = 0.30, 95% CI 0.02–1.47, *t* = 2.07, *P* = 0.04) were both significantly associated with an increase in adolescent problem-solving. There was no significant association between treatment group and changes in dyadic conflict or adolescents’ involvement	Potential limitations of study include: Small sample size Homogeneous sample of adolescents Exclusive focus on mother–adolescent interactions Findings specific to adolescents with depression who respond to psychosocial treatment
Gunlicks-Stoessel and Mufson,^39^ 2016, pilot RCT based in the USA, lasting 16 weeks	15 adolescents with mean age 15.2 years, meeting DSM-IV diagnostic criteria for MDD, MDD and dysthymic disorder or depressive disorder not otherwise specified Gender: Female - 86.7% Ethnicity: Latino 93.3%, White 86.7%, biracial 6.7%, Black 6.7% Modal family income: $25 000–$39 000 20% of participants had comorbid anxiety disorder	IPT-AP: *n =* 9 Focus on understanding parent–adolescent relationship and communication patterns, and teaching and practising communication and relationship-building skills Up to 14 sessions over 16 weeks Family involved for eight sessions (two were parent only, six were combined parent–adolescents): 57% of total time	IPT-A: *n =* 6 Individual therapy with parents joining for part of the first session to receive psychoeducation about depression and IPT-A, and part of the last session to discuss relapse prevention 12 45-min sessions over a 16-week course Average family involved for 1.5 sessions: 67.5 min and 12.5% of total time	CBQ - parent and adolescent report: Mother's behaviour Mother–adolescent dyadic behaviour Father's behaviour Father–adolescent dyadic behaviour Adolescent's behaviour Significant decrease in CBQ adolescent report on mothers’ conflict behaviour (*t*(14) = 2.43, *P* = 0.029), mother–adolescent dyadic behaviour (*t*(14) = 4,00, *P* = 0.001) and fathers’ conflict (*t*(14) = 3.09, *P* = 0.008) behaviour in both treatment conditions. IPT-AP resulted in significantly lower scores for CBQ adolescent report on fathers’ behaviour (F = 3.77, *P* < 0.10, η^2^ = 0.24), and CBQ mother report on mother–adolescent dyadic behaviour (F = 4.82, *P* < 0.05, η^2^ = 0.29) compared with IPT-A	
Kolko et al,^40^ 2000, three-arm RCT based in the USA, lasting 24 months	103 adolescents with mean age 15.6 (s.d. 1.4) years, meeting DSM-III-R diagnostic criteria for MDD Gender: Female 75% Ethnicity: White 85% Socioeconomic status: Mean Hollingshead 40.2 (s.d. 13.1) 16% of participants had comorbid oppositional disorder 32% of participants had comorbid anxiety 37% of participants had comorbid dysthymia 37% of participants were suicidal at intake	SBFT: Combined aspects of functional family therapy, intended to clarify concerns and identify dysfunctional patterns of family interaction, and a behavioural family systems approach, aimed at teaching communication and problem-solving skills to reduce family conflict 12–16 weekly sessions plus two to four booster sessions for those with clinical need Family involved for all sessions. Up to 25% of total treatment time (4 h) could be allocated to patient individually without family	CBT: Intervention focused heavily on cognitive restructuring, also utilising behavioural activation, and taught problem-solving skills on a case-by-case basis NST: Focused on providing support, aiding the adolescent in affect identification and expression of feelings, and discussing patient-initiated options for addressing problems. NST therapists relied on accurate empathy and reflexive listening, and refrained from giving advice, setting limits or teaching specific skills 12–16 weekly sessions Up to 25% of total treatment time (4 h) could be allocated to family involvement	CBQ: assess conflict and negative communication ACQ: evaluates parent–child relationships across specified problem areas FAD: measure family functioning, including general functioning, problem-solving, communication, roles, affective responsiveness, affective involvement and behaviour control Acute effects (end of 6 weeks): No significant treatment × time interaction found for adolescent-report CBQ or FAD. CBT (*χ*^2^(1) = 11.60, *P* < 0.0007) and SBFT (*χ*^2^(1) = 7.84, *P* < 0.005) had a greater effect than NST on general functioning and behaviour control for parent-reported FAD. Long-term effects (end of 24 months): No treatment × time interaction for adolescent-report CBQ or FAD. SBFT and NST resulted in greater improvement over time than CBT on parent-reported CBQ and ACQ	Potential limitations of study include: Measurement of specific domains may have been limited, e.g. because of using self-report instruments Other factors that were not assessed after the trial had ended may have contributed to the findings obtained at the end of the follow-up period Measurement of treatment-specific domains may have been confounded by depression
Sanford et al,^43^ 2006, unblinded RCT based in Canada, lasting 9 months	31 adolescents with mean age 15.9 (s.d. 1.4) years, meeting DSM-IV diagnostic criteria for MDD Gender (% female): TAU group 11%, TAU + FPE group 9% Socioeconomic status: Total family income <$25 000 9.7% Comorbid diagnosis: psychosis 12.9%, dysthymic disorder 19.3%, separation anxiety disorder 12.9%, social phobia 61.3%, generalised anxiety disorder 45.1%, OCD 22.6%, PTSD 16.1%	TAU + FPE: Aims to increase family knowledge about adolescent depression, increase understanding of the experience of depression and its impact on the family, to strengthen family communication, to enhance effective coping, problem-solving and management of crisis and relapses 12 90-min sessions over 6 months plus a booster 2–3 months later Family involved for all sessions: 18 h and 100% of total time	TAU: Consisting of individual or group counselling and/or drug therapy (first line: SSRI, venlafaxine, bupropion, in combination with anxiolytics or antipsychotics if indicated) with supportive case management Family involvement not reported	SSAI: parent–adolescent subscale, used to measure adolescent social functioning FAD: general functioning subscale, used to measure family functioning Adjective checklist: used to measure adolescent relationships with mother and father Group trajectories were significantly different for the following outcomes: negative adjectives score for adolescent–mother relationship, positive and negative adjective scores for adolescent–father relationship and adolescent global social functioning SSAI scores according to both adolescent and parent informants. FPE group moved further than controls toward more positive functioning and relationships. No significant difference between group trajectories for FAD general functioning, or for positive adjectives score for adolescent–mother relationship	Potential limitations of study include: Small sample size Participants not blinded to intervention versus control Fidelity to the FPE intervention self-rated by therapists rather than through independent observations Baseline differences between treatment groups in global social functioning and overall functioning despite randomisation. Experimental group was functioning worse than controls Psychosocial and drug treatments were not standardised. Those receiving FPE were more likely than controls to continue antidepressant medication throughout the study
Mufson et al,^42^ 1999, RCT based in the USA, lasting 12 weeks	48 adolescents with mean age 15.7 (s.d. 1.4) years in IPT-A group and 15.9 (s.d. 1.7) in control group meeting DSM-III-R diagnostic criteria for MDD Gender (% female): IPT-A group 75%, control group 70.8% Socioeconomic status: Public assistance, mother: IPT-A group *n* = 10; control group *n* = 11 Comorbid diagnosis: IPT-A group: dysthymic disorder 29%, anxiety disorder 88%; control group: dysthymic disorder 13%, anxiety disorder 88%	IPT-A: Addresses common adolescent development issues, e.g. separation from parents, exploration of authority in relationships to parents, initial experience with the death of a relative or friend, peer pressure and single-parent families. Weekly 45 min sessions for 12 weeks with up to three additional sessions Extent of family involvement unclear, likely one session	Clinical monitoring: Therapists given a brief treatment manual asking them to refrain from advice giving or skills training and to use the sessions to review depressive symptoms, school attendance, assess suicidality and listen supportively Monthly 30 min sessions for 12 weeks with up to one additional session per month No family involvement	SAS-SR: Brief self-report instrument with separate category for family IPT-A-treated patients reported significantly better functioning compared with control patients for their overall level of functioning (F_(1,44)_ = 7.1, *P* = 0.01), functioning with their friends (F_(1,44)_ = 5.8, *P* = 0.02) and functioning in dating relationships (F_(1,44)_ = 5.9, *P* = 0.02). IPT-A-treated adolescents showed significant better skills at week 12 on positive problem-solving orientation (*t*_21_ = −2.4, *P* < 0.05) and rational problem-solving (*t*_21_ = −2.4, *P* < 0.05)	Potential limitations of study include: Small sample size Substantial attrition from the control condition Use of self-report measures of social functioning
Lewinsohn et al,^41^ 1990, three-arm RCT based in the USA, lasting 12 weeks	59 adolescents with mean age of 16.26 (s.d. 1.17) years in adolescent and parent group, 16.15 (s.d. 0.98) years in adolescent only group and 16.28 (s.d. 1.17) years in control group meeting DSM-III diagnostic criteria for MDD (49%), minor depression (7%) or intermittent depression (44%) Mean age (all groups): 16.23 years Gender (% female): adolescent and parent group 52.6%, adolescent only group 61.9%, control group 68.4%	CWD-A and separate parent group: Cognitive–behavioural psychoeducational group for adolescents (see Comparator description). Separate parent group with goal to provide an overview of skills and techniques taught in adolescent group sessions in an effort to promote parental acceptance and reinforcement of the expected positive changes in their teenagers. Parents also presented with coping skills to address family problems without resorting to arguments or fights 14 2-h CWD-A sessions for adolescents only, over 7 weeks Seven 2-h parent sessions over 7 weeks (14 h)	CWD-A: Skills-training-orientated treatment sessions with focus on teaching methods of relaxation, increasing pleasant events, controlling irrational and negative thoughts, increasing social skills and conflict-resolution component including communication and problem-solving skills. 14 2-h CWD-A sessions over 7 weeks No family involvement Waitlist control: Participants were informed of their treatment status and offered a referral to another treatment agent if they felt they could not wait for treatment. At the conclusion of the waiting period participants in this condition completed the post-assessment measures and subsequently participated in a treatment group - 0 sessions over 7 weeks - 0 family involvement	Issues Checklist Parent-report and adolescent-reported measure of conflict containing a list of 44 issues that are often problematic for adolescents and parents No significant difference in Issues Checklist scores between treatment groups in both parent- and adolescent-reported measures	Potential limitations of study include: Limited generalisability of results because the sample was being actively recruited and comprised only older adolescents without psychiatric comorbidity, whose parents were willing to be involved in treatment
Bernal et al,^37^ 2019, RCT based in Puerto Rico, lasting 1 year	121 adolescents aged 13–17.5 years meeting DSM-IV diagnostic criteria for MDD (49%), minor depression (7%) or intermittent depression (44%) No age data Gender (% female): intervention group 40%, control group 67.2% Socioeconomic status: public school: intervention group 62.3%, control group 63.3% Psychiatric comorbidity: intervention group 65%, control group 55.7%	CBT + TEPSI: TEPSI integrates aspects of cognitive and interpersonal theories and is designed to teach parents about signs of depression, and effective ways to help their adolescent cope with their depressed mood states. Sessions included didactic material, practice exercises, and personal or family projects to be completed between sessions. The first session includes information about symptoms, causes and myths of depression in adolescents; the next three sessions are dedicated to cognitive strategies; the final four sessions focus on interpersonal skills 12 CBT sessions for adolescents only, over 12 weeks Eight 2-h TEPSI sessions over 12 weeks (16 h)	Individual CBT: Culturally adapted individual CBT intervention modified for adolescents, based on concepts of behavioural and cognitive therapy, cognitive therapy and rational-emotive therapy. Attempts to identify thoughts and actions that influence mood with the goal to diminish depressive feelings, teach methods of preventing depression, and increase participant's sense of control over their life 12 weekly CBT sessions No family involvement	Brief Family Assessment Measure: self-report scale consisting of 14 statements designed to measure overall family functioning Family Emotional Involvement and Criticism Scale (FEICS): 14-item scale measuring perceived criticism and emotional involvement in the family from a family member's perspective Familism Scale: 14-item scale measuring values and attitudes toward the family, focusing on identification and attachment between family members and feelings of family loyalty and reciprocity. Main effect observed for family involvement (regression coefficient –0.150, s.e. = 0.058, estimated s.e. = -2.581, *P* = 0.010), but not perceived criticism, subscales of the FEICS. CBT only group showed significant increase in perception of family emotional involvement compared with the CBT + TEPSI group. CBT only group showed significant increase in perception of familism compared with the CBT + TEPSI group, where perception of familism decreased.	Potential limitations of study include: Specific needs of the family were not addressed; thus, although the TEPSI condition provided relevant information, it may not have influenced critical mechanisms of change in MDD

RCT, randomised controlled trial; MDD, major depressive disorder; SBFT, systematic behavioural family therapy; CBT, cognitive–behavioural therapy; NST, nondirective supportive therapy; IPT-AP, interpersonal psychotherapy for adolescents and parents with depression; IPT-A, individual interpersonal psychotherapy for adolescents with depression; CBQ, Conflict Behaviour Questionnaire; ACQ, Areas of Change Questionnaire; FAD, Family Assessment Device; TAU, treatment as usual; TAU + FPE, treatment as usual plus family psychoeducation; OCD, obsessive compulsive disorder; PTSD, post-traumatic stress disorder; SSRI, selective serotonin reuptake inhibitor; SSAI, Structured Social Adjustment Interview; SAS-SR, Social Adjustment Scale - self-report version; CWD-A, Coping with Depression Course for Adolescents; TEPSI, CBT plus parent psychoeducational intervention; FEICS, Family Emotional Involvement and Criticism Scale.

Five studies included measures of comorbid anxiety.^37–40,42,43^ However, only one study included analyses involving anxiety.^40^ There was significant variation in the interventions used to improve family-focused communication across studies, which included systematic behavioural family therapy (SBFT),^38,40^ interpersonal psychotherapy (IPT) for adolescents and parents with depression,^39^ treatment as usual plus family psychoeducation,^43^ IPT for adolescents with depression,^42^ coping with depression course for adolescents and a separate parent group,^41^ and cognitive–behavioural therapy (CBT) plus parent psychoeducational intervention.^37^ Because of the variation across studies, there was considerable heterogeneity in the structure of the interventions. For example, some interventions only included parents in a single 45 min session,^42^ whereas in others parents attended every session (18 h in total^43^).

### Effectiveness of family-focused interventions at improving symptoms of anxiety and depression

Several of the studies found evidence that family-focused interventions reduced depressive symptoms (β = 1.02, *P* < 0.001;^37^ *P* < 0.001;^39^ *P* < 0.001;^41^ *P* < 0.05^42^) and major depressive disorder diagnoses (β = –1.11, *P* < 0.001;^37^ χ^2^ = 9.41, *P* < 0.01;^41^ *P* < 0.02^42^). However, a caveat is that these studies found similar improvements to depressive symptoms and major depressive disorder diagnosis when using interventions that did not focus on family involvement (^37^; η^2^ = 0.00, *P* > 0.10;^39^ major depressive disorder: χ^2^ = 0.001, *P* > 0.05^41^) or did not include a comparative intervention.^42^ One study did, however, find that the family-focused intervention led to better parent-reported outcomes on adolescents’ depressive symptoms relative to an intervention without a focus on family communication (*P* < 0.01^41^), although these differences were no longer present at a 6-month follow-up. In contrast, one study found that an intervention that did not include a focus on family communication was better than an intervention focused on family communication at reducing symptoms of depression (CBT: *P* = 0.003, SBFT: *P* = 0.99^38^). A study that compared treatment as usual versus treatment as usual with family psychoeducation found no effect of familial involvement on depressive symptoms or major depressive disorder diagnosis (*P* = 0.052^43^). Therefore, these studies do not provide clear evidence that family-focused interventions are more effective at improving symptoms of depression relative to interventions without a focus on family communication.

The one study that did include analyses of anxiety symptoms found that CBT was more effective than a family-focused intervention at improving symptoms at a 24-month follow-up.^40^

#### Key information on family communication measures

The majority of communication measures included in our review were self-report (*n* = 7); only one measure of communication was recorded by observing interactions between adolescents and their parent. Also, several studies (*n* = 3) included both adolescent and adult reports of communication. Four studies only included either adolescent or parent reports. The most commonly used measure was the Conflict Behaviour Questionnaire (*n* = 2), which assesses conflict and negative communication between adolescents and their parents. We recommend future research aims to develop measures that capture the subjective experience of communication, along with objective patterns of communication between family members, and that such measures are completed by both adolescents and their parents.

### Do interventions aimed at improving communication work, for whom do they work, in what contexts and why?

Although the studies reviewed provide limited evidence for the effectiveness of family-focused interventions in improving symptoms of anxiety and depression, there was significant variation across studies in how communication was measured and the features of communication treated as outcome measures (Table 1). Although this may reflect the multifaceted way in which we presume communication to affect mental health, this heterogeneity means that we have structured our results to address what features of communication are improved by psychological intervention. Once we have identified the features of communication that are amenable to intervention, we can establish for whom these interventions work, in what contexts and why.

#### Interventions examining familial involvement

Family involvement describes the degree to which the adolescent feels able to communicate their emotions to their family and the degree to which they perceive their family's communication toward them to be critical. Drawing on Fitzpatrick and Ritchie's conceptualisation of communication in families, this feature of communication could reflect the extent to which families deploy emotional resources toward one another.^7^ This feature of communication was examined by three studies,^37,38,40^ which provided limited evidence that family-focused interventions improved perceptions of familial involvement. Bernal et al^37^ found that adolescents assigned to a CBT-only group that did not involve family reported greater family emotional involvement after the intervention, whereas adolescents that completed CBT with additional parent psychoeducation reported no change in family emotional involvement (*P* < 0.05, effect size 0.77). Two studies^38,40^ found that neither SBFT, CBT or nondirective supportive therapy (NST) was associated with changes in familial involvement from pre- to post-intervention. SBFT is a therapy that intends to clarify concerns and identify dysfunctional patterns of family communication, teaching communication and problem-solving skills, whereas NST aims to provide support for adolescents to identify their feelings and consider options to address their issues (see Table 1). However, one study^43^ found a significant difference in the trajectories of affective involvement, with adolescents who completed treatment as usual plus family psychoeducation reporting greater familial involvement compared with those who only completed treatment as usual (*P* < 0.05).

#### Interventions examining problem-solving

Problem-solving reflects the aspect of family communication that describes how the family shares responsibility for solving daily emotional and social crises.^7^ Three studies examined adolescents’ problem-solving communication behaviours as outcomes (i.e. behaviours where adolescents generated solutions to interpersonal problems).^38,40,42^ Two studies found that family-focused interventions improved problem-solving abilities relative to interventions without a focus on family involvement (β = 0.30, *P* = 0.04;^38^ *P* < 0.05^42^), whereas one study found no difference between interventions with and without a focus on family involvement.^40^ Although Dietz et al^38^ found that completing SBFT improved problem-solving in adolescent–mother dyads relative to CBT and NST, Kolko et al^40^ found no difference between SBFT, CBT and NST on problem-solving immediately after the intervention and at a 24-month follow-up, despite the emphasis in SBFT on teaching problem-solving skills. Notably, Dietz et al^38^ coded adolescent–mother dyads, whereas Kolko et al^40^ used a self-report measure completed by parents and adolescents. Of note, the findings by Dietz et al^38^ were rated as having low risk of bias in the measurement of outcomes, whereas Kolko et al^40^ had a high risk of bias in their measurement of outcomes. CBT does, however, include psychoeducational content on problem-solving, which may improve interpersonal problem-solving skills.^40^ Although two of the three included studies found evidence that family-focused interventions improved adolescents’ problem-solving skills, these studies suffered from a high overall risk of bias. Therefore, we suggest that the strength of evidence for the effectiveness of family-focused interventions at improving problem-solving skills is weak, based on the studies included in this review.

#### Interventions examining conflict behaviour

Familial conflict is an aspect of communication that reflects the inverse of receptivity to new information, as described by Fitzpatrick and Ritchie.^7^ The four studies examining conflict behaviour between adolescents and their parents^38–41^ provided mixed evidence regarding the efficacy of family-focused interventions for improving conflict behaviours. Participants who completed IPT for adolescents and parents with depression reported less adolescent–father conflict (reported by adolescents; *P* < 0.100, η^2^ = 0.24) and adolescent–mother conflict (reported by mothers; *P* < 0.050, η^2^ = 0.29) relative to individual IPT.^39^ Consistent with these findings, parents of adolescents who completed SBFT reported greater improvements to dyadic behaviour compared with parents of adolescents who completed CBT at 24 months follow-up (*P* < 0.001, χ^2^ = 12.64^40^), although similar improvements were found for participants who completed NST relative to CBT in this study.^40^ Two further studies did not find a difference between interventions with or without a family-focused component on conflict behaviour.^38,41^

#### Interventions examining family functioning

Three studies examined general family functioning,^37,40,43^ which describes the organisational properties of families and patterns of transactions between family members. For example, measures of general family functioning ask how responsive family members are toward the emotions of other family members, and how accepted the individual feels within the family dynamic.^44^ Communication is integral to measures of family functioning, as they focus on verbal ways in which issues are resolved within the family (e.g. talking to people directly rather than going through go-betweens).^8^ These studies provided limited evidence that family-focused interventions improved general family functioning to a greater extent than interventions without a family-focused component. Although one study found a family-focused intervention improved family functioning relative to NST (χ^2^ = 12.64, *P* < 0.007^40^), improvements to general family functioning were similar between treatments with or without a family-focused component.^37,40,43^ Therefore, although family-focused interventions may improve general family functioning, there is an absence of evidence to suggest this improvement is greater than interventions that do not explicitly include families.

#### Interventions examining social adjustment

Two studies examined social adjustment,^42,43^ which describes the extent to which individuals adjust to social roles (i.e. professional or educational roles, social and leisure activities, and role within the family^45^). Poor adjustment to social rules can lead to friction, and measures of social adjustment ask how well the individual is able to communicate to others around them in their role (e.g. as the child of their parent).^46^ These studies suggested that family-focused interventions were effective at improving adolescents’ social adjustment, as both studies reported greater social functioning scores after completing a family-focused intervention compared with treatment as usual (*d* = 0.93–0.96^43^) or clinical monitoring (*P* = 0.01^42^). However, these studies did not compare a family-focused intervention to another psychotherapeutic intervention. Therefore, although family-focused interventions appear successful at improving social adjustment, we cannot assess whether they are more successful than other types of interventions.

## Discussion

The current systematic review examined the efficacy of family-focused interventions to improve communication within families for adolescents with anxiety disorders and/or depression. Across the seven studies reviewed, we found mixed evidence regarding the effectiveness of family-focused interventions to improve any facet communication within families, at least compared with existing interventions that do not include families within the intervention. Yet, we were struck by the absence of high-quality research into improving communication in families of young people with anxiety disorders and/or depression. Our systematic literature search yielded a small number of highly heterogeneous studies, which, despite being randomised controlled trials, had a high risk of bias (Table 2).^36^ Therefore, in answer to the question, ‘Do family-focused interventions improve communication within families, for whom does this work, in what contexts, and why?’, our team of experts by lived experience, researchers and clinicians suggest that there is insufficient evidence to provide an authoritative answer to this question and encourage further research on this important topic.

**Table 2:** Cochrane Risk of Bias 2 tool

Risk of Bias						
	A	B	C	D	E	F
Dietz et al^38^ 2014	?	−	−	+	?	−
Gunlicks-Stoessel and Mufson^39^ 2016	?	+	−	−	?	−
Kolko et al^40^ 2000	?	+	−	−	?	−
Sanford et al^43^ 2006	?	+	−	?	?	?
Mufson et al^42^ 1999	?	+	+	−	?	−
Lewinsohn et al^41^ 1990	?	−	−	+	?	−
Bernal et al^37^ 2019	?	+	−	−	?	−

A represents bias arising from the randomisation process, B represents bias owing to deviations from intended interventions, C represents bias owing to missing outcome data, D represents bias in measurement of the outcome, E represents bias in selection of the reported result and F represents overall bias.

### Do family-focused interventions improve communication within families within context of anxiety disorders and depression?

We found substantial variation in the ways in which family communication was conceptualised (as conflict, family functioning, familial involvement or problem-solving). This heterogeneity prevented us from drawing firm conclusions about whether improving family communication is an active ingredient in the treatment of anxiety disorders and/or depression in 14- to 24-year-olds. However, most of the studies found that family-focused interventions did not lead to significant improvements in features of communication relative to existing psychotherapeutic interventions. Although this could be interpreted to suggest that family-focused interventions do not improve communication, we instead propose that in context of the significant limitations of the included studies (which we discuss below), there is insufficient evidence to conclude whether family-focused interventions can improve communication within families. Indeed, this perspective was reflected by our advisory group, who all agreed that communication within families was a topic worthy of further study in the context of anxiety disorders and depression. There was some promising evidence that communication can be improved (relative to treatment as usual/waitlist), but the mixed findings, heterogeneous measurement and non-specificity of the results (e.g. compared with other treatments) make it impossible to recommend an approach to improving communication at this stage.

### For whom do family-focused interventions work?

We believe that a conceptual shift is required to advance our understanding of for whom improving communication in families works. The analogy from physical health – of the accepted importance of addressing high blood pressure – is useful here in at least two ways. First, high blood pressure is itself a risk factor for other health problems (such as hardened arteries, which, in turn, are a risk factor for heart failure). Second, effective treatment of high blood pressure can be a pre-requisite for other medical interventions to be conducted safely (such as before elective surgery). Ineffective communication might similarly be a non-specific risk factor for common mental health problems, as indicated by the multifaceted way in which studies have linked poor family communication to mental health outcomes,^22,23,27,32^ and therefore may be an appropriate target for prevention. Further, for interventions to be effective, communication within families might need to be addressed as a pre-requisite for some individuals. For example, as one of the members of our YPAG stated ‘effective communication is really important. Without it, young people, who may require only very minimal support to reduce their anxiety, can't get that fulfilled’. Indeed, the inability to express the need for support is consistent with empirical evidence that poor communication with parents can create a barrier to the access of treatment.^34^

### In what context do family-focused interventions work?

We are also unfortunately unable to draw conclusions about how to best target communication in psychological therapy. There was significant heterogeneity in the interventions used to deliver family-focused content (including CBT, family psychoeducation, IPT for adolescents with depression and SBFT) and often embedded in programmes with significant additional content. A number of these interventions are time-limited and highly structured, established for individual delivery rather than delivery to adolescent with their parents (e.g. CBT, IPT for adolescents with depression). As such, we raise the question of whether content aimed at improving communication should be integrated within, and therefore potentially replace or shorten, existing treatment programme elements or be the focus of a separate and distinct intervention; and, in either case, how should this be practically implemented?

Four studies in our review included a family-focused component to an intervention that traditionally did not involve family members.^37,39,41,43^ Of these studies, only one found the addition of the family-focused intervention improved communication (specifically conflict behaviour^39^). In this study, an adaption of IPT for adolescents with depression was delivered with parents attending several sessions. Given the existing emphasis of IPT for adolescents with depression on communication,^47^ it may be that some treatments are more amenable to the inclusion of family-focused content compared with interventions that focus on other mechanisms of change (e.g. cognitive restructuring in CBT). Indeed, the missed potential for family-focused interventions to benefit adolescents was highlighted by one study that found participants in classical CBT reported greater feelings of family emotional involvement compared with participants in CBT supplemented with a family-focused component.^37^ One interpretation of this finding is that increased parental involvement following a family-focused intervention may be incongruent with adolescents’ desire for increased autonomy from caregivers,^48^ producing adverse outcomes. Certainly, care needs to be taken with adding elements to existing evidence-base interventions, as it may inadvertently reduce the therapy's effectiveness by incurring a kind of opportunity cost. Furthermore, a key question that we hoped to address but could not, is when a focus on communication might be indicated or not; further research is urgently needed to establish this.

### Strengths and limitations

The included studies were all randomised controlled trials, with all but one^43^ utilising blinded allocation to the treatment condition when compared with a control condition. Furthermore, three studies compared the family-focused intervention to another intervention and a control condition,^38,40,41^ which provided stronger evidence for the efficacy (or lack thereof) of family-focused interventions.

However, we also identified several limitations in the studies reviewed. Of critical importance is the small sample size in half of the studies included in the review,^38,39,42,43^ meaning these studies most likely did not have statistical power to identify differences between treatment arms. Furthermore, there was a disproportionate focus on adolescent–mother dyads, either because of an explicit design choice^38^ or fathers not attending as often.^37,39^ If the reason for poor family communication was a result of adolescent–father conflict, this could be one possible explanation for the absence of evidence regarding the efficacy of family-focused interventions at improving communication. This view was endorsed by our advisory group who suggested that it is the ‘underlying dynamics [of the family] that need to be looked at’.

Finally, our focus was limited to children and young people with diagnoses of anxiety disorders and/or depression as part of the project to assess active ingredients in the treatment of these disorders.^49^ Thus, we were unable to examine the importance of addressing family communication in the face of a more general sense of severe emotional distress. This important issue was emphasised by our experts by lived experience:

‘… it was clear that there was more emotional distress that went unrecognised and untreated [in child and adolescent mental health services]. It wasn't until DBT [dialectical behaviour therapy] skills were offered at aged 18+ (in adult services), which directly addressed communication skills, that both my daughter and I benefitted from greatly improved communication.’

### Recommendations for policy makers, clinicians and funders

In the light of our findings, the theoretical and practical importance of communication, and our advisory group discussions, we call for funders to prioritise studies that will develop measures that capture essential features of communication. One such feature, emphasised by our experts by lived experience, is that ‘Communication is not clear cut, straightforward, it can be a way of connecting, rather than a way of putting some message across.’ Thus, affective dimensions like connection must be captured in addition to definitions that rely on the transmission of information. We do not expect this to be simple. Indeed, as another expert by experience explained, ‘Effective communication is more than just exchanging information. It's about understanding the emotions and intention behind the information. That's the bit that's hard to measure. There's much more going on, especially in families.’ Consistent with this view, empirical studies have demonstrated that discrepancies between the adolescent's and parents’ perceptions of the effectiveness of their communication with one another are associated with greater internalising problems.^50^ Therefore, the objective act of exchanging information may not be sufficient to measure communication. Rather, we propose that measures should be developed that capture the affective experience of connecting through verbal exchanges to examine communication within families.

In conclusion, it is important to acknowledge limitations of the current systematic review. Although our definition of family communication was guided by theoretical work on this topic^7,16^ and was endorsed by our advisory group of lived experience experts, these theoretical definitions did not map exactly onto the outcome measures used in the included studies. Indeed, this issue further emphasises the need for the development of new tools to measure family communication that reflect both theory and the lived experience of communication within families.

Communication is of central theoretical and practical importance to young people's mental health, yet we have found an absence of evidence about the role of improving family communications as an active ingredient in the treatment of anxiety and/or depression in young people aged 14–24 years. As a team of clinicians, experts by experience and scientists, we call for future studies to be designed to conceptualise communication more rigorously, to capture young people's lived experience of what communication is; to identify how to improve communication within families and to better understand for which young people and families this will be most beneficial. As stated by a member of our advisory group:

‘If you get it wrong at the foundational stage, if young people don't feel that they can speak openly and be heard and validated for their experiences, then that's a really shaky start and where do you get that if it doesn't start in the family home?’

## Supporting information

Lloyd et al. supplementary material Lloyd et al. supplementary material

## Data Availability

The data that support the findings of this study are available from the corresponding author, P.J.L., upon reasonable request.
