# Supplementary material for: The impact of family interventions on communication in the context of anxiety and depression in those aged 14–24 years: systematic review of randomised control trials
Source: BJPsych Open. 2023 Aug 29;9(5):e161. doi: 10.1192/bjo.2023.545 (PMC10594091; doi:10.1192/bjo.2023.545)
Supplement: Lloyd et al. supplementary material [file S2056472423005458sup001.docx]

**Supplementary Materials**

**Supplementary Table 1: Full search strategy for the electronic databases**

MEDLINE (05.01.2022)

| 1 | Child/ | |
| --- | --- | --- |
| 2 | Child* | |
| 3 | Young adj1 (people OR person OR adult) | |
| 4 | Teen* | |
| 5 | Adolescent/ | |
| 6 | Adolescen* | |
| 7 | Youth | |
| 8 | 1-7 OR | **N=4207046** |
| 9 | Anxiety/ OR Anxiety Disorder/ | |
| 10 | Anxi* OR Anxiety Disorder OR GAD OR Generali#ed Anxiety Disorder | |
| 11 | Depression/ | |
| 12 | Depress* OR Depressive Disorder | |
| 13 | Common mental disorder OR Common mental problem OR Common mental health disorder OR Common mental health problem OR CMD | |
| 14 | 9-13 OR | **N=757455** |
| 15 | (Family OR carer OR guardian OR parent* OR family/) adj2 (intervention OR programme OR therapy OR adaptation OR support OR awareness OR advice OR counselling) OR family therapy/ | **N = 167164** |
| 16 | Communication OR relationship* OR interaction OR family conflict/ OR family relations/ | |
| 17 | (interpersonal OR social OR language) adj1 (functioning OR skills) | |
| 18 | 16 OR 17 | **N = 3221493** |
| 19 | Randomized controlled trial.pt. | |
| 20 | Controlled clinical trial.pt. | |
| 21 | Randomized.ab | |
| 22 | Placebo.ab. | |
| 23 | Drug therapy.fs. | |
| 24 | Randomly.ab. | |
| 25 | Trial.ab | |
| 26 | Groups.ab. | |
| 27 | OR/19-26 | |
| 28 | Exp animals/ not humans.sh | |
| 29 | 27 NOT 28 | **N=4543059** |
| 30 | AND/8, 14, 15, 18, 29 | **N=829** |

EMBASE (05.01.22)

| 1 | Child/ | |
| --- | --- | --- |
| 2 | Child* | |
| 3 | Young adj1 (people OR person OR adult) | |
| 4 | Teen* | |
| 5 | Adolescent/ | |
| 6 | Adolescen* | |
| 7 | Youth | |
| 8 | 1-7 OR | **N=3981185** |
| 9 | Anxiety/ OR Anxiety Disorder/ | |
| 10 | Anxi* OR Anxiety Disorder OR GAD OR Generali#ed Anxiety Disorder | |
| 11 | Depression/ | |
| 12 | Depress* OR Depressive Disorder | |
| 13 | Common mental disorder OR Common mental problem OR Common mental health disorder OR Common mental health problem OR CMD | |
| 14 | 9-13 OR | **N=1094190** |
| 15 | (Family OR carer OR guardian OR parent* OR family/ OR extended family/) adj2 (intervention OR program* OR therap* OR adaptation OR support OR awareness OR advice OR counsel?ing) OR family therapy/ OR family centered care/ OR family counseling/ OR family service/ | **N = 157782** |
| 16 | Communication OR relationship* OR interaction OR family attitude/ OR family conflict/ OR family coping/ OR family functioning/ | |
| 17 | (interpersonal OR social OR language) adj1 (functioning OR skills) | |
| 18 | 16 OR 17 | **N = 3948031** |
| 19 | Randomized controlled trial | |
| 20 | Controlled clinical trial | |
| 21 | Randomized | |
| 22 | Placebo | |
| 23 | Drug therapy | |
| 24 | Randomly | |
| 25 | Trial | |
| 26 | Groups | |
| 27 | OR/19-26 | |
| 28 | Exp animals/ not humans | |
| 29 | 27 NOT 28 | **N=890568** |
| 30 | AND/8, 14, 15, 18, 29 | **N=40** |

PsychInfo

| 1 | Child* | |
| --- | --- | --- |
| 2 | Young adj1 (people OR person OR adult) | |
| 3 | Teen* | |
| 4 | Adolescen* | |
| 5 | Youth | |
| 6 | 1-5 OR | **N=1239992** |
| 7 | Anxiety/ OR Anxiety Disorders/ | |
| 8 | Anxi* OR Anxiety Disorder OR GAD OR Generali#ed Anxiety Disorder | |
| 9 | Major depression/ | |
| 10 | Depress* OR Depressive Disorder | |
| 11 | Common mental disorder OR Common mental problem OR Common mental health disorder OR Common mental health problem OR CMD | |
| 12 | 7-11 OR | **N=548413** |
| 13 | ((Family OR carer OR guardian OR parent* OR family/ OR extended family/) adj2 (intervention OR program* OR therap* OR adaptation OR support OR awareness OR advice OR counsel?ing)) OR family therapy/ OR family intervention/ OR family work relationship/ | **N = 110989** |
| 14 | Communication OR relationship* OR interaction OR family conflict/ OR family relations/ | |
| 15 | (interpersonal OR social OR language) adj1 (functioning OR skills) | |
| 16 | 14 OR 15 | **N = 1295983** |
| 17 | Randomized controlled trial | |
| 18 | Controlled clinical trial | |
| 19 | Randomized | |
| 20 | Placebo | |
| 21 | Drug therapy | |
| 22 | Randomly | |
| 23 | Trial | |
| 24 | Groups | |
| 25 | OR/17-24 | |
| 26 | Exp animals/ not humans | |
| 27 | 25 NOT 26 | **N=829583** |
| 28 | AND/6, 12, 13, 16, 27 | **N=1314** |

CENTRAL

| 1 | MeSH descriptor: [Child] explode all trees | |
| --- | --- | --- |
| 2 | Child* | |
| 3 | Young NEXT (people OR person OR adult) | |
| 4 | Teen* | |
| 5 | MeSH descriptor: [Adolescent] explode all trees | |
| 6 | Adolescen* | |
| 7 | Youth | |
| 8 | 1-7 OR | **N=334985** |
| 9 | MeSH descriptor: [Anxiety] explode all trees | |
| 10 | Anxi* OR Anxiety Disorder OR GAD OR Generalised Anxiety Disorder | |
| 11 | MeSH descriptor: [Depression] explode all trees | |
| 12 | Depress* OR Depressive Disorder | |
| 13 | Common mental disorder OR Common mental problem OR Common mental health disorder OR Common mental health problem OR CMD | |
| 14 | 9-13 OR | **N=141842** |
| 15 | (Family OR carer OR guardian OR parent*) NEAR/2 (intervention OR program* OR therap* OR adaptation OR support OR awareness OR advice OR counselling) | |
| 16 | MeSH descriptor: [Family Therapy] explode all trees | |
| 17 | 15 OR 16 | **N=12964** |
| 18 | Communication OR relationship* OR interaction | |
| 19 | MeSH descriptor: [Family Conflict] explode all trees | |
| 20 | MeSH descriptor: [ Family Relations] explode all trees | |
| 21 | (interpersonal OR social OR language) NEAR1 (functioning OR skills) | |
| 22 | 18-21 OR | **N = 157124** |
| 34 | AND/8, 14, 17, 22 | **N=1212**  **Limit to Trials = 860** |
